# Evaluation of Cardiovascular Risk Factors after Hepatitis C Virus Eradication with Direct-Acting Antivirals in a Cohort of Treatment-Naïve Patients without History of Cardiovascular Disease

**DOI:** 10.3390/jcm11144049

**Published:** 2022-07-13

**Authors:** Diego Casas-Deza, Ana Martínez-Sapiña, Silvia Espina, Beatriz Garcia-Rodriguez, Eva M. Fernandez-Bonilla, Alejandro Sanz-Paris, Yolanda Gonzalez-Irazabal, Vanesa Bernal-Monterde, Jose M. Arbones-Mainar

**Affiliations:** 1 Gastroenterology Department, Miguel Servet University Hospital, 50009 Zaragoza, Spain; diegocasas8@gmail.com (D.C.-D.); silespina@gmail.com (S.E.); evaferbo@yahoo.es (E.M.F.-B.); 2 Instituto de Investigación Sanitaria (IIS) Aragon, 50009 Zaragoza, Spain; bea_garcia_rodriguez@hotmail.com (B.G.-R.); sanzparisalejandro@gmail.com (A.S.-P.); yolgonira@gmail.com (Y.G.-I.); 3 Clinical Microbiology Department, Miguel Servet University Hospital, 50009 Zaragoza, Spain; amartinezsa@salud.aragon.es; 4 Clinical Biochemistry Department, Miguel Servet University Hospital, 50009 Zaragoza, Spain; 5 Nutrition Department, Miguel Servet University Hospital, 50009 Zaragoza, Spain; 6 Translational Research Unit, Miguel Servet University Hospital, Instituto Aragonés de Ciencias de la Salud (IACS), 50009 Zaragoza, Spain; 7 Centro de Investigación Biomédica en Red Fisiopatología Obesidad y Nutrición (CIBERObn), Instituto Salud Carlos III, 28029 Madrid, Spain

**Keywords:** HCV, cholesterol, lipids

## Abstract

Background: Hepatitis C virus (HCV) produces changes at multiple levels in host metabolism, especially in lipid profile and cardio-metabolic risk. It is unclear how HCV eradication by direct-acting antivirals (DAAs) modifies those changes. Objective: To evaluate the impact of DAA treatment on different risk factors associated with cardiovascular disease. Methods: Prospective study with two-year follow-up. All patients treated with DAAs in the Liver Clinic of a tertiary hospital were included. Patients co-infected with HBV or HIV, with other causes of liver disease, on lipid-lowering treatment, pregnant, or with previous HCV treatment were excluded. The results were analyzed using linear mixed models. Results: 167 patients (53% female, 9.6% cirrhosis) were included. Low plasma lipid levels were observed before initiating HCV eradication. During the first year after treatment with DAA, we observed a sustained increase in cholesterol, triglycerides, HDL cholesterol (only in men), and LDL-cholesterol levels. An ameliorated glycemic control was also observed with a decrease in fasting insulin and reduced HOMA. Iron metabolism and coagulation function also improved with lower levels of serum ferritin and prothrombin activity; these biochemical changes resulted in a new diagnosis of hypercholesterolaemia in 17.4% of patients, requiring initiation of statins in 15%. Two non-fatal cardiovascular events were observed during the first 2 years of follow-up. Conclusions: DAA treatments returned plasma lipids to the normal range without increasing either the occurrence of cardiovascular events or the consumption of lipid-lowering medication beyond what is normal in a sex- and age-matched population.

## 1. Introduction

The Hepatitis C Virus (HCV) is an RNA virus that is one of the main causes of liver morbidity and mortality. It is estimated that in the world there are about 71 million infected people, with a worldwide incidence of 1.75 million new cases per year [1]; however, HCV does not cause a direct cytopathic effect on host cells and most of its extrahepatic manifestations of chronic infection are likely related to the virus-mediated alteration of host metabolisms, such as immune responses and several metabolic pathways [2].

Ample evidence indicates that HCV interferes with carbohydrate and lipid metabolism, ultimately causing liver steatosis, insulin resistance (IR), and cardiovascular disease (CVD) [3]. Elevated circulating homocysteine, as well as alterations in iron metabolism, through their association with inflammation and oxidative stress, are also considered independent risk factors for CVD (reviewed in Ref. [4]). Not surprisingly, individuals with HCV showed significantly higher serum levels of homocysteine than non-infected controls [5]. Alterations in vitamin B12 and folate pathways, both required for homocysteine remethylation into methionine, are also affected during HCV infection [6]; likewise, elevated serum ferritin and iron levels have been common findings in patients with HCV since described by Di Bisceglie et al. [7]. Lastly, there is also considerable evidence that HCV is able to activate hemostasis through several mechanisms (reviewed in Ref. [8]) creating a prothrombotic state which ultimately can increase the risk of future cardiovascular events.

Initially, therapeutic options for HCV eradication were based on combinations of pegylated interferon and ribavirin, with low response rates [9]; however, the approval of the first direct action antiviral (DAA) in 2013 was supposed an extraordinary breakthrough in antiviral therapy, reaching rates of sustained viral response (SVR) close to 100% [10,11]. The whole concept of the disease has been modified and, although not completely eliminated, the risk of all-cause mortality and hepatocellular carcinoma (HCC) development has been now substantially reduced [11,12]; this has also shifted the focus from the fight for patients‘ survival to preventing the long-term effects of the HCV-related metabolic complications beyond the virologic cure.

DAA treatments decrease liver inflammation, improve transaminase levels and reduce liver fibrosis progression [13]; however, HCV eradication produces a simultaneous increase in serum cholesterol and LDL levels [14], creating a combination of circumstances that might aggravate the risk of atherosclerosis and CVD. The results of the different studies are, conversely, quite often contradictory, since some show increases in HDL cholesterol levels [15,16,17,18], but this is not reported in other studies [19,20,21] or even a decrease is observed [21]. In the case of triglycerides, disparate results have also been reported, with minimal or absent changes [17,22] or even decreases [14,23]. The virological cure seems to produce an improvement in IR, although there is no evidence of its long-term effect or whether it only occurs in specific populations [24,25]. On the other hand, DAA treatments seem to reduce iron and ferritin levels [26], while their effects on homocysteine metabolism have not been evaluated in prospective studies. Reports regarding the impact of DAA on the coagulation function have shown a reversal of hypercoagulability in patients with HCV-related cirrhosis [27] or an improvement of both the individual pro- and anti-coagulants with a net effect that does not substantially modify their balance [28].

We hypothesize that those disparate results might be explained, at least partially, because all these studies have been carried out in populations with different racial or ethnic statuses, different DAA treatment schemes, and different proportions of patients with liver cirrhosis; from 25% to 80% [29,30] or coinfection with HIV, present in up to the 60% in some studies [19]; moreover, sex differences in the prevalence and burden of CVD may also account for some of the above disparities [31]. Accordingly, this study sought a holistic approach with a complete and simultaneous evaluation of different risk factors associated with CVD. From the baseline, through the DAA treatments up to 2-year follow-up. As a secondary aim, we investigated possible sex differences regarding those risk factors and their longitudinal trajectories.

## 2. Materials and Methods

### 2.1. Ethical Considerations

The clinical research ethics committee of Aragon (CEICA) evaluated and approved the project with study code PI17/0390. Informed consent was collected from all patients who agreed to participate in the study.

### 2.2. Design and Selection Criteria

This is a single-centre, prospective, longitudinal, non-interventional study carried out in the hepatology outpatient department of the Miguel Servet University Hospital, a tertiary hospital in Zaragoza (Spain). The design is a before-after study, in which each patient is their own control, assessing changes before and after treatment.

All treatment-naive patients with a confirmatory serological diagnosis of chronic HCV infection who had been referred to hepatology clinics for evaluation of treatment with DAA were consecutively recruited. Patients co-infected with human immunodeficiency virus (HIV) and/or hepatitis B virus (HBV) were excluded. Patients with previous alterations in their lipid or under pharmacological treatment (hypercholesterolemia, hypertriglyceridemia) were also excluded. Pregnant patients and those previously treated for HCV were also excluded. Patients with other causes of liver disease, such as cholestatic diseases, Wilson’s disease, hemochromatosis, or autoimmune hepatitis were also excluded. Patients with heavy alcohol use were excluded too. Finally, patients with severe neuropsychiatric comorbidity that prevented proper follow-up were also excluded.

Alcohol consumption was defined as consumption of at least 20 g/day for men and 10 g/day for women, without reaching a risky intake. Heavy alcohol use was defined as consumption of >40 g/day for men and/or >25 g/day for women or >28 units per week for men and >17 units per week for women and/or ≥6 units per occasion for males and ≥4 units per occasion for females [32].

Four visits were made throughout the study. During the initial (baseline) visit, personal and family history, as well as anthropometric variables were collected by interview with the patient. All patients underwent a non-invasive assessment of liver fibrosis by transient elastography (FibroScan 430 Mini, Echosens, France). For an elastography measurement to be considered valid, it had to have an IQR < 30% and a percentage of valid measurements above 60%. Liver cirrhosis was defined as a transient elastography score of >14 kPa, or liver biopsy showing Metavir fibrosis 4, or clinical evidence of liver cirrhosis.

In all four visits, serum samples were obtained to assess biochemical parameters. After the baseline visit, the remaining determinations were made at the end of antiviral treatment, at 12 weeks after the end of treatment (SVR 12), and at 48 weeks after the end of treatment (SVR 48). Subsequently, a clinical follow-up was performed two years after antiviral treatment. Biochemical determinations were performed at the Clinical Biochemistry Department in the Miguel Servet University Hospital at four follow-up points (baseline, end of treatment, 12 weeks after the end of treatment, and 48 weeks after the end of treatment). All extractions were performed after 8 h of fasting by the patient. Variables related to liver function and damage (AST, ALT, GGT, FA, bilirubin, pre-albumin and albumin), iron profile (iron, ferritin, and transferrin saturation index), vitamin B12 metabolism (homocysteine and vitamin B12), glycemic profile (glucose, insulin, HOMA-IR), blood count, and coagulation were assessed. The serological indices FIB-4 [33] and APRI [34] as non-invasive markers of fibrosis were calculated. Additionally, a study of lipid metabolism, including triglycerides (TG), total cholesterol (CHOL), LDL-cholesterol, HDL-cholesterol, apolipoprotein A (apoA) and apolipoprotein B (apoB) was performed.

During follow-up, the occurrence of major cardiovascular events (nonfatal stroke, nonfatal myocardial infarction, and cardiovascular death) was assessed, as well as the initiation of lipid-lowering medications (statins, ezetimibe, bile acid sequestrants, and fibrates). We used the cohort Sanitas Data4Good to investigate the prevalence of lipid-lowering drugs in a representative cohort of the population from Spain; this cohort collects clinical records, medications, and pre-existing conditions prior to hospital admission.

### 2.3. Statistical Analysis

Results are presented as means and SDs for normal variables or medians and interquartile ranges (IQRs) for non-normal data. For the longitudinal investigation, we used an intent-to-treat (ITT) approach as some patients did not complete their follow-up according to protocol. All DAA-treated patients were included in the analyses regardless of subsequent drop-outs before the conclusion of the study. The data were modeled using linear mixed models (LMM) to (1) take into consideration the repeated assessment of each variable and (2) account for the effects of covariates on the variables’ change over time. Those LMM models were, hence, adjusted for age and protease inhibitors intake and produced different *p*-values which captured the variation over time of each variable for the entire cohort (p_long_), overall sex differences (p_sex_), and sex-specific longitudinal changes (the interaction between longitudinal changes and sex; p_long*sex_). Statistical analysis was carried out in R 4.1.2. and the appropriate packages.

## 3. Results

**Figure 1:**
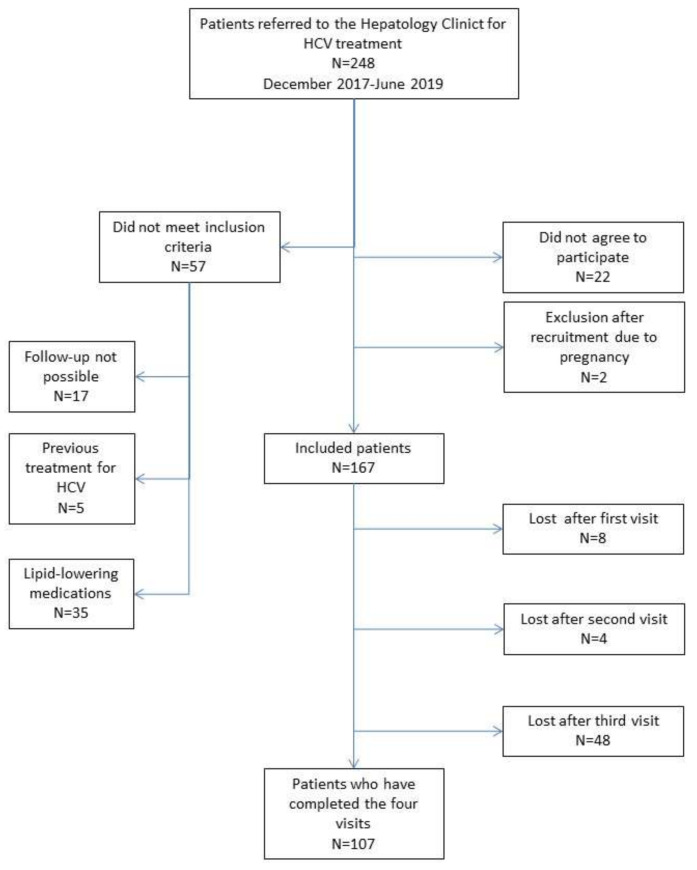
Patients’ flowchart.

Between December 2017 and June 2019, 248 patients received antiviral treatment with DAAs at our institution, of whom 167 met the inclusion criteria and were included in the study. The patients’ flowchart is shown in Figure 1.

### 3.1. Baseline Characteristics

All patients were treatment–naïve and their characteristics are described in Table 1. Individuals’ ages ranged from 21 to 88 years and women were slightly older than men (57.3 y. vs. 53.3 y. on average respectively; *p* = 0.039). The most frequent viral genotype was 1, present in 70.9% of the patients. Characteristics related to HCV infection can be found in Table 2.

More than half of the patients had a low degree of fibrosis (F0–F1) and only 16 had liver cirrhosis (9.58%). Of the 16 patients with liver cirrhosis, 7 had portal hypertension, all of them with esophageal varices. Only one had ascites at the time of treatment. As for the total cohort, only 2 patients (1.20%) had a history of cardiovascular events. In terms of cardiovascular risk factors, 51 patients (30.5%) had some factor, the most prevalent being hypertension (22.2%), followed by diabetes (4.19%). Only 18% of patients were smokers at the time of the study and 13.8% of the cohort had some alcohol consumption at the time of treatment.

All patients were treated with DAA combinations: 56.9% with drugs with protease inhibitors (Glecaprevir/Pibrentasvir or Elbasvir/Grazoprevir) and 43.1% with drugs without protease inhibitors (Sofosvuvir/Velpatasvir or Sofosbuvir/Ledipasvir). The overall sustained virological response (SVR was 100%)

### 3.2. Longitudinal Hepatic Changes

At the 1-year follow-up, there was an overall reduction in mean total bilirubin (p_long_ < 0.001) (Table 3); however, a sex-specific effect showed an ~20% decrease in men, while women only experienced a 5% decrease in total bilirubin (p_long*sex_ = 0.032). We also observed an increase in albumin and pre-albumin during the follow-up (both p_long_ < 0.001). Again, a sex-specific effect occurred, and men had an ~25% increase in pre-albumin while women only experienced a 5% increase in total pre-albumin (p_long*sex_ = 0.002).

Liver function tests significantly improved from baseline to SVR48 irrespective of sex. Phosphatase alkaline steadily decreased during the follow-up (p_long_ < 0.001) while we observed an abrupt ~3-fold reduction in the transaminase values occurring mainly during DAAs treatments (p_long_ < 0.001 for GGT, ALT, and AST); this steep reduction paralleled the fibrosis scores based on laboratory parameters, APRI and FIB-4, which also showed a significant early improvement (both p_long_ < 0.001) during the treatment.

### 3.3. Longitudinal Extra-Hepatic Changes

**Figure 2:**
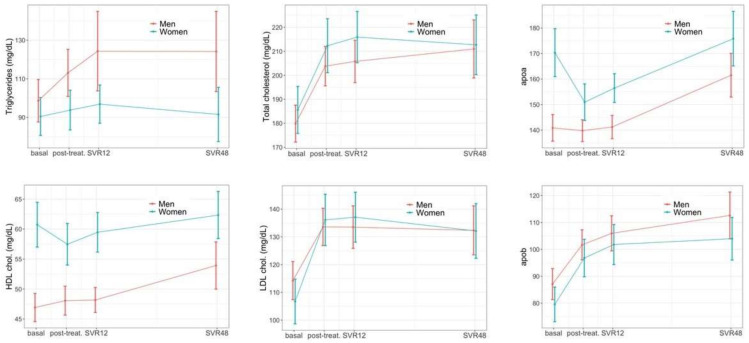
Lipid metabolism changes during follow-up.

The average levels of triglycerides (TG), total cholesterol (CHOL), LDL-cholesterol, HDL-cholesterol, apoA and apoB rose significantly during the follow-up for the entire cohort (p_long_ = 0.003 for TG and p_long_ < 0.001 for CHOL, LDL, HDL, apoA and apoB) (Figure 2 and Supplementary Table S1); this elevation was clearly visible at the end of treatment already for CHOL and LDL and was maintained for the entire follow-up (Figure 2). Compared to men, women had consistently lower levels of TG (p_sex_ = 0.023) and increased HDL (p_sex_< 0.001) throughout the follow-up. We observed an interaction between sex and longitudinal changes by which the magnitude of the increase in HDL, from the basal analysis to the SVR48, was greater in men than in women (15% vs. 2.6% respectively, p_long*sex_ = 0.018).

**Figure 3:**
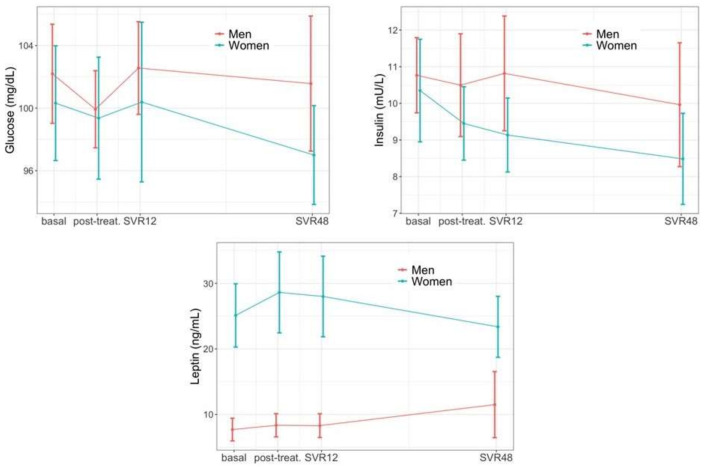
Glycemic profile changes during follow up.

Figure 3 illustrates that during 1-year of follow-up neither glucose nor leptin presented significant changes compared to baseline (p_long_ = 0.077) but we did observe a reduction of insulin upon DAA treatments (p_long_ = 0.004) which translated to an improved homeostatic model assessment (HOMA-IR) for the entire cohort (p_long_ = 0.006). Women consistently presented greater values of leptin (p_sex_ < 0.001) and reduced HOMA (p_sex_ = 0.045) over the course of the study, while none of these parameters presented sex-specific changes in their longitudinal trajectories.

**Figure 4:**
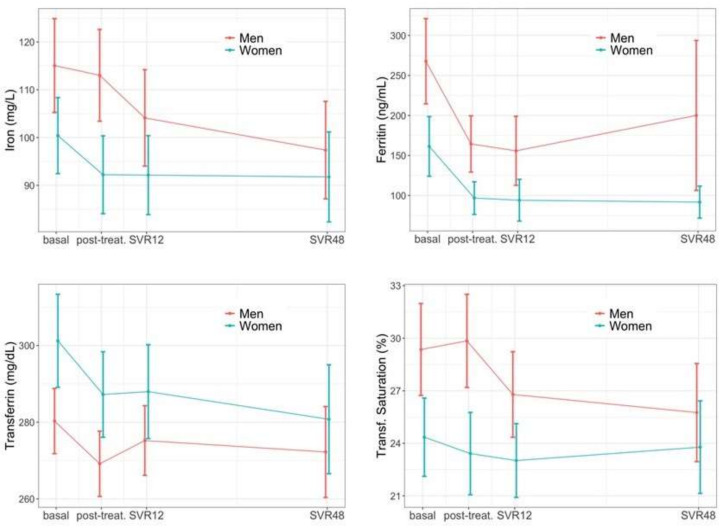
Iron metabolism changes during follow-up.

The levels of serum iron, ferritin and transferrin saturation decreased significantly during follow-up (all p_long_ < 0.001), while transferrin was unaffected (Figure 4). It is worth noting that, compared to men, women had significant increased transferrin (p_sex_ = 0.003) and reduced values of serum iron (p_sex_ = 0.005), ferritin (p_sex_ < 0.001), and transferrin saturation (p_sex_ < 0.001) throughout the follow-up. We also observed a sex-specific effect in the magnitude of the reduction in the transferrin saturation (14% vs. 2.1% for men and women, respectively, p_long*sex_ = 0.025).

**Figure 5:**
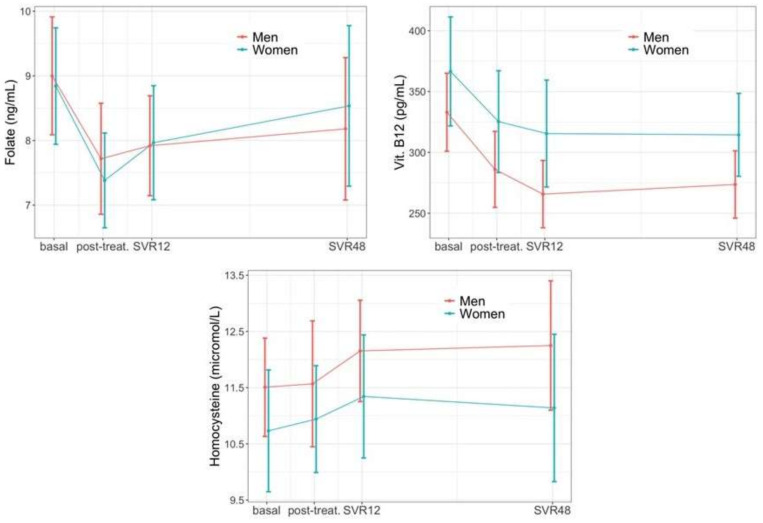
Vitamine B12, folate and homocysteine changes during follow-up.

During the follow-up, there was an overall reduction in folic acid (p_long_ = 0.029) and vitamin B12 (p_long_ < 0.001) accompanied by an increase in homocysteine (p_long_ = 0.016). None of these parameters presented sex-specific changes in their longitudinal trajectories (Figure 5).

Next, we sought to evaluate the effects of DAA on coagulation changes in our cohort. The international normalized ratio (INR), partial thromboplastin time (PTT), prothrombin activity (PA), and derived fibrinogen changed significantly during the follow-up for the entire cohort (all p_long_ < 0.001); however, those changes occurred at different times depending on the studied variables. Thus, derived fibrinogen increased during DAA treatments while INR started to decline at SVR12 mirroring the increase in TTP and prothrombin activity. No sex-specific changes for those variables were observed in their longitudinal trajectories.

**Figure 6:**
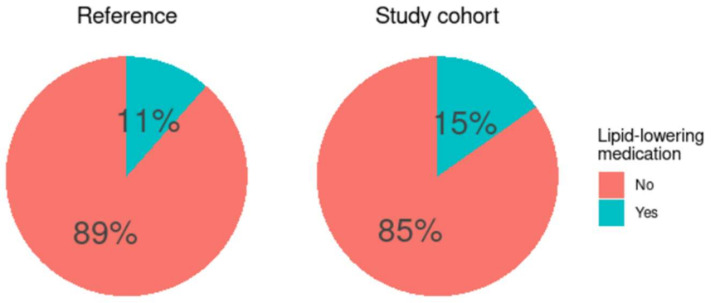
Comparison of the usage of lipid-lowering medication between the study cohort and a reference cohort.

Cardiovascular events and initiation of lipid-lowering medications were assessed 2 years after DAA treatments. Two patients had non-fatal cardiovascular events (1 acute myocardial infarction and 1 stroke) and 25 patients (15%) had started lipid-lowering treatments during the 2-year follow-up. Finally, we investigated whether the use of these medications was greater than expected in the general population. For that, our study cohort was sex- and age-matched (1:3) with a large cohort of the hospitalized population from Spain in which drug usage before admission had been recorded (Figure 6). We did not observe significant differences between both cohorts in the percentage of individuals on lipid-lowering medication (*p* = 0.264).

## 4. Discussion

In this study, DAAs for HCV eradication in treatment-naive patients was associated with increased plasma lipids and homocysteine, improved glycemic control and iron homeostasis, as well as reduced prothrombin activity during the first year post-treatment, and these changes occurred in both sexes. We observed 2 new non-fatal cardiovascular events during the first 2 years of follow-up of a cohort of 167 individuals with a median age of 55.2 years.

The advent of DAAs, with cure rates of 95–100%, changed the disease paradigm. Despite its very high effectiveness in eradicating the infection, the effect of this treatment and the cure of the virus on the host metabolism remains an issue that is not fully clarified. Several studies have tried to assess the impact of DAA treatment on host metabolism, with very mixed results. Most of the studies conducted were retrospective, which is an important limitation. In addition, the inclusion of special populations (HIV co-infected patients, cirrhotic patients, transplant recipients, etc.) may lead to biased results.

Therefore, our study sought to identify as purely as possible the effect of antiviral treatment and eradication of HCV infection. For this reason, co-infected, transplanted or non-naive patients, as well as those on lipid-lowering treatments were excluded from the study. Although it was not an exclusion criterion, the proportion of patients with advanced liver disease in our cohort is very low (9.6%). Furthermore, our study is one of the studies with the longest follow-up (1 year analytically and 2 years clinically); this makes it possible to verify the temporality of the changes observed in the early stages of follow-up. Early changes might be a direct effect of DAA treatments, while medium- and long-term changes might be explained by the HCV eradication and liver status amelioration.

Alterations in the lipid profile of HCV-infected patients have been clearly established; these changes are due to the interaction between HCV and host cholesterol synthesis pathways. The virus hijacks these pathways for its own replicative cycle [35,36], thus causing changes in the patient’s basal metabolism; these alterations in lipid metabolism lead to an increased risk of CVD in HCV-infected patients, with increased morbidity and mortality from extra-hepatic causes [37,38]. Recently, HCV eradication by DAAs translated into a significant reduction of cardiovascular events in a prediabetic population [39] as well as in a large cohort with a high prevalence of advanced fibrosis (F3–F4: 70.5%) [40]. In our study, with a low prevalence of diabetes and advanced fibrosis, all lipid parameters increased after HCV eradication. Total cholesterol and LDL-cholesterol showed the earliest and most pronounced increase. Because of its importance and its occurrence right after DAA treatments, this phenomenon has been the easiest to identify in all studies, including those that are retrospective, with short follow-ups, or with heterogeneous populations.

On the other hand, triglycerides and HDL cholesterol showed less abrupt and more gradual increases. Furthermore, in both cases, a sex-dependent effect was observed, with the increase being mainly in men. Taking into account HDL, these results were in line with those observed by Inoue et al. [17], Gonzalez-Colominas et al. [22], and Shimizu et al. [41], all long follow-up studies. Interestingly, studies that reported no differences, such as those from Cheng et al. [42], Jain et al. [43], and Ichikawa et al. [44] had reduced follow-ups (<6 months). As for triglycerides, only the studies by Doyle et al. [45] and Cheng et al. [39] observed an increase in serum levels. Again, it is likely that the different methodologies of the studies have influenced the disparity in the results of the previous studies, although this parameter was not assessed in many of the studies. Most of the studies in which no changes in triglyceride levels were observed have reduced sample sizes and those with larger sample sizes had a significant percentage of HIV co-infected patients [21,46]. HIV antiretroviral treatments may cause significant changes in triglyceride levels, which may have masked changes due to HCV treatment.

The changes in glucose and insulin metabolism that we observed in our study were similar to those previously described in the literature [47,48,49]. Although we did not see a reduction in serum glucose levels, we did see a reduction in insulin levels. These changes were reflected in an improved HOMA-IR index, which is consistent with a recent report of significant reductions in HOMA-insulin resistance and an increase in HOMA-insulin sensitivity following HCV clearance by DAA treatment [50]; however, as with lipid metabolism, some studies have found no such changes [25,45,46], which can be explained, at least partially, by a genotype-dependent effect whereby an improvement in carbohydrate metabolism occurs in those patients infected with HCV genotype 1 [51]. In our cohort ~70% of the patients were infected with this genotype, which may explain why we observed these changes.

The relationship between vitamin B12 and liver disease has been previously described [52,53,54]. Several studies have postulated elevated B12 levels as a possible marker of advanced liver disease or hepatocarcinoma [55,56]; this relationship has also been observed in patients with chronic HCV infection and the interaction of vitamin B12 with the HCV replication cycle has been described [6]. In these patients, higher B12 levels were associated with more advanced levels of liver fibrosis. Our results show a marked decrease in B12 levels after DAA treatment paralleling the improvement of liver damage, reflected in the decrease of liver damage markers during follow-up and the improvement of APRI and FIB-4 indices, as it has been previously described [57]. In a similar way to vitamin B12, iron metabolism is modified by HCV infection but also influences its replication cycle [58]. HCV infection reduces hepcidin levels, which alters iron metabolism [59]; moreover, ferritin is an inflammatory marker elevated during chronic infection [60]. Our results showed a change in the ferric profile, with a generalized normalization of the parameters, especially ferritin.

To summarize our main findings, chronic HCV infection reduced plasma lipids, and both cholesterol and triglyceride levels increased after HCV eradication; however, this increase should be considered as a return to normal values according to sex and age. Thus, median values of total cholesterol were 210 and 215 mg/dL for men and women respectively in our cohort after 1-year follow-up; these values are in line with those of 206 and 214 mg/dL for men and women in the age range of 45–64 years, as reported in the ENRICA study [61], one of the largest national population data on plasma lipid in Spain. In addition, although 15% of patients started lipid-lowering treatments, this percentage is not different from the prevalence of lipid-lowering medication use among the general population matched by age and sex. In our opinion, the prevention of cardiovascular risk in these patients is essential. Close surveillance should be implemented in patients with previous cardiovascular risk factors or with alterations during follow-up. We propose that such monitoring should probably be performed by the primary care physician and integrated into the routine follow-up of the patient.

Our work has, in our view, several strengths. The first two are its prospective design and the large sample size. In addition, it is a cohort without co-infections (neither HBV nor HIV), with strict selection criteria and a very low percentage of patients with cirrhosis; moreover, the 2-year follow-up time is also very important, as this has allowed long-term changes to be assessed. It can be argued that 2 years may be too short to demonstrate any meaningful changes in cardiovascular outcomes; however, two recent studies reported a significant reduction of cardiovascular events after HCV clearance by DAAs with median follow-ups of 24 and 28 months [39,40]. Weaknesses include loss to follow-up and the absence of biopsy assessment of fibrosis. Other limitations are the observational nature of the study which prevents establishing a cause–effect relationship between DAA therapy and the observed results. In addition, the study is monocentric, which may lead to a bias in the selection of patients.

## 5. Conclusions

Eradication of HCV with DAAs causes long-lasting changes in the patient’s lipid, glycaemic and ferric profiles, as well as in vitamin B12 metabolism, even in an otherwise healthy cohort of patients; these changes were not trivial because they led to the start of new treatments in almost a fifth of the patients; however, we should note that DAA treatments returned plasma lipids to the normal range without increasing the occurrence of cardiovascular events beyond normal in an age-matched population.

## Figures and Tables

**Table 1:** Clinical status and patient background.

Variable	All n = 167	Men n = 88	Women n = 79	*p*-Value
Age	55.2 (12.4)	53.3 (10.8)	57.3 (13.6)	0.039
Personal history of CVD	3 (1.80%)	2 (2.28%)	1 (1.27%)	1.000
Stroke	2 (1.20%)	1 (1.14%)	1 (1.27%)	1.000
Ischemic cardiopathy	0 (0%)	0 (0%)	0 (0%)	1.000
Peripheral vasculopathy	1 (0.60%)	1 (1.14%)	0 (0.00%)	1.000
Family history of CVD				0.091
None	122 (73.1%)	69 (78.4%)	53 (67.1%)	
Stroke	2 (1.20%)	1 (1.14%)	1 (1.27%)	
Ischemic cardiopathy	30 (18.0%)	10 (11.4%)	20 (25.3%)	
Peripheral vasculopathy	13 (7.78%)	8 (9.09%)	5 (6.33%)	
CV risk factors	51 (30.5%)	26 (29.5%)	25 (31.6%)	0.900
Hypertension	37 (22.2%)	19 (21.6%)	18 (22.8%)	1.000
Diabetes Mellitus	7 (4.19%)	5 (5.68%)	2 (2.53%)	0.448
Smoking				0.077
Never	84 (50.3%)	37 (42.0%)	47 (59.5%)	
Previously	30 (18.0%)	19 (21.6%)	11 (13.9%)	
Current	53 (31.7%)	32 (36.4%)	21 (26.6%)	
Alcohol use	23 (13.8%)	16 (18.2%)	7 (8.86%)	0.128
Body mass index (kg/m^2^)	25.0 [22.6;27.1]	25.0 [23.4;27.1]	24.9 [22.0;27.3]	0.748
Abdominal perimeter (cm)	92.3 (11.2)	93.3 (10.1)	91.3 (12.3)	0.446
Liver fibrosis (kPa)	6.60 [5.40;10.2]	7.00 [5.50;11.3]	6.35 [5.15;8.80]	0.141
Liver fibrosis (Metavir)				0.384
1	87 (53.4%)	41 (48.2%)	46 (59.0%)	
2	26 (16.0%)	13 (15.3%)	13 (16.7%)	
3	27 (16.6%)	16 (18.8%)	11 (14.1%)	
4	23 (14.1%)	15 (17.6%)	8 (10.3%)	
Liver cirrhosis				0.670
No	151 (90.4%)	79 (89.8%)	72 (91.1%)	
Yes, without PHT	9 (5.39%)	6 (6.82%)	3 (3.80%)	
Yes, with PHT	7 (4.19%)	3 (3.41%)	4 (5.06%)	
Esophageal varices	7 (4.19%)	4 (3.41%)	3 (3.80%)	1.000
Ascites	1 (0.60%)	1 (1.14%)	0 (0.00%)	1.000

CVD: Cardiovascular Disease; CV: cardiovascular; PHT: Portal Hypertension.

**Table 2:** HCV infection characteristics and treatment details.

Variable	All n = 167	Men n = 88	Women n = 79	*p*-Value
Treatment				0.909
Sofosbuvir/Velpatasvir	66 (39.5%)	36 (40.9%)	30 (38.0%)	
Ledipasvir/Sofosbuvir	6 (3.59%)	3 (3.41%)	3 (3.80%)	
Glecaprevir/Pibrentasvir	65 (38.9%)	35 (39.8%)	30 (38.0%)	
Elbasvir/Grazoprevir	30 (18.0%)	14 (15.9%)	16 (20.3%)	
Treatment length				0.848
12 weeks	96 (57.8%)	52 (59.1%)	44 (56.4%)	
8 weeks	70 (42.2%)	36 (40.9%)	34 (43.6%)	
Viral genotype				0.352
1	5 (3.03%)	4 (4.65%)	1 (1.27%)	
1a	50 (30.3%)	29 (33.7%)	21 (26.6%)	
1b	62 (37.6%)	31 (36.0%)	31 (39.2%)	
2	4 (2.42%)	1 (1.16%)	3 (3.80%)	
2a/c	2 (1.21%)	0 (0.00%)	2 (2.53%)	
3	16 (9.70%)	8 (9.30%)	8 (10.1%)	
3a	2 (1.21%)	0 (0.00%)	2 (2.53%)	
4	21 (12.7%)	10 (11.6%)	11 (13.9%)	
4c/d	2 (1.21%)	2 (2.33%)	0 (0.00%)	
5	1 (0.61%)	1 (1.16%)	0 (0.00%)	
Viral load (log)	6.05 [5.36;6.54]	6.11 [5.62;6.53]	5.89 [5.16;6.55]	0.199

**Table 3:** Hepatic changes from baseline to SVR48.

	Men				Women						
Variable	Basal n = 87	post-T n = 81	SVR12 n = 80	SVR48 n = 51	Basal n = 79	post-T n = 78	SVR12 n = 75	SVR48 n = 56	p_long_	p_sex_	p_long*sex_
Bilirubin (mg/dL)	0.74 [0.68;0.79]	0.65 [0.59;0.70]	0.63 [0.58;0.69]	0.60 [0.53;0.67]	0.65 [0.58;0.73]	0.59 [0.54;0.65]	0.58 [0.52;0.65]	0.62 [0.54;0.71]	<0.001	0.006	0.032
Albumin (g/dL)	4.25 [4.17;4.32]	4.28 [4.21;4.35]	4.34 [4.28;4.41]	4.34 [4.22;4.46]	4.11 [4.04;4.18]	4.12 [4.06;4.18]	4.17 [4.10;4.23]	4.26 [4.19;4.34]	<0.001	0.002	0.611
Pre-albumin (mg/dL)	22.3 [21.0;23.7]	26.2 [24.7;27.6]	27.1 [25.7;28.5]	28.1 [26.0;30.1]	18.4 [17.2;19.7]	20.4 [19.3;21.5]	21.7 [20.6;22.9]	22.0 [20.5;23.5]	<0.001	<0.001	0.002
Alkaline phosphatase (U/L)	84.7 [79.7;89.6]	85.0 [79.7;90.4]	82.3 [77.3;87.3]	79.2 [74.2;84.2]	93.0 [85.9;100]	91.5 [84.8;98.2]	88.8 [81.8;95.8]	89.8 [82.2;97.4]	<0.001	0.175	0.523
GGT (U/L)	91.9 [66.6;117]	29.1 [24.1;34.2]	30.5 [24.9;36.2]	36.4 [27.0;45.7]	60.3 [46.5;74.1]	26.3 [20.0;32.6]	25.8 [20.9;30.7]	23.9 [19.7;28.2]	<0.001	0.023	0.255
AST (U/L)	63.0 [51.7;74.3]	26.9 [24.3;29.4]	26.1 [23.7;28.4]	27.2 [23.1;31.2]	62.6 [43.1;82.2]	25.1 [22.1;28.1]	24.5 [19.7;29.4]	22.7 [20.8;24.7]	<0.001	0.379	0.786
ALT (U/L)	81.7 [65.8;97.5]	23.9 [20.3;27.5]	21.5 [18.7;24.4]	24.5 [17.7;31.4]	62.7 [45.7;79.7]	20.9 [16.3;25.6]	19.3 [12.4;26.2]	15.8 [14.0;17.5]	<0.001	0.036	0.447
APRI	1.08 [0.73;1.42]	0.44 [0.36;0.51]	0.41 [0.33;0.49]	0.39 [0.30;0.47]	1.07 [0.59;1.55]	0.41 [0.31;0.50]	0.37 [0.27;0.47]	0.33 [0.27;0.38]	<0.001	0.465	0.772
FIB-4	2.54 [2.03;3.06]	1.92 [1.67;2.17]	1.92 [1.66;2.17]	1.76 [1.52;2.01]	2.99 [2.23;3.76]	2.13 [1.73;2.54]	2.07 [1.72;2.43]	2.06 [1.66;2.46]	<0.001	0.922	0.636

Data are shown as the median and interquartile range (95% confidence interval); post-T: post-treatment, SVR12: 12 weeks after treatment, SVR 48: 28 weeks after treatment, APRI: AST to Platelet Ratio Index, FIB-4: Fibrosis-4 Index.

## Data Availability

The data presented in this study are available on request to the corresponding author with prior authorization of our Ethical Committee that can be obtained at https://www.iacs.es/investigacion/comite-de-etica-de-la-investigacion-de-aragon-ceica/ceica-evaluaciones-y-otras-presentaciones (accessed on 10 May 2022).

## Supplementary Materials

The following supporting information can be downloaded at: https://www.mdpi.com/article/10.3390/jcm11144049/s1, Table S1: Serum lipid concentrations during follow up.
