# Supplementary material for: Evaluation of Cardiovascular Risk Factors after Hepatitis C Virus Eradication with Direct-Acting Antivirals in a Cohort of Treatment-Naïve Patients without History of Cardiovascular Disease
Source: J Clin Med. 2022 Jul 13;11(14):4049. doi: 10.3390/jcm11144049 (PMC9315656; doi:10.3390/jcm11144049)
Supplement: Supplementary file 1 [file jcm-11-04049-s001.zip › jcm-1745889-supplementary.pdf]

Supplementary Table S1. Serum lipid concentrations during follow up

Variable	Basal	End of treatment	SVR	1 year after end of treatment	P
**Triglycerides (mg/dl)**	94.7 [87.3;102]	104 [95.5;112]	111 [99.3;123]	107 [94.5;120]	0.003
**Total cholesterol (mg/dl)**	183 [176;189]	208 [201;215]	211 [204;218]	212 [203;220]	<0.001
**LDL-cholesterol (mg/dl)**	111 [105;116]	135 [129;140]	135 [129;141]	132 [126;139]	<0.001
**HDL-cholesterol (mg/dl)**	53.5 [51.1;55.9]	52.7 [50.5;54.9]	53.6 [51.5;55.7]	58.3 [55.5;61.2]	<0.001
**Non-HDL cholesterol (mg/dl)**	129 [124;135]	155 [149;162]	157 [151;163]	154 [146;161]	<0.001
**LDL/HDL cholesterol ratio**	3.20 [2.70;4.30]	3.70 [3.20;4.40]	3.80 [3.10;4.80]	3.70 [3.20;4.60]	<0.001
**Apolipoprotein A (mg/dl)**	155 [149;161]	145 [141;149]	149 [145;152]	169 [162;176]	<0.001
**Apolipoprotein B (mg/dl)**	83.5 [79.2;87.8]	99.3 [94.9;104]	104 [99.1;109]	108 [102;114]	<0.001

SVR: Sustained viral Response
